# MDCT Morphometry of the Thoracolumbar Junction in a Turkish Cohort: Surgical, Radiological, and Forensic Implications

**DOI:** 10.3390/tomography12080109

**Published:** 2026-07-28

**Authors:** Lale Duman, Esra Yamansavcı Şirzai

**Affiliations:** 1 Department of Radiology, University of Health Sciences, Dr. Suat Seren Chest Diseases and Thoracic Surgery Training and Research Hospital, Izmir 35120, Turkey; 2 Department of Thoracic Surgery, University of Health Sciences, Dr. Suat Seren Chest Diseases and Thoracic Surgery Training and Research Hospital, Izmir 35120, Turkey

**Keywords:** thoracolumbar junction, morphometry, Turkish population, multi-detector computed tomography, rib anomalies, pedicle morphometry, forensic anthropology

## Abstract

**Background**: The thoracolumbar junction (TLJ) includes the T11–L2 vertebral segments. It is a pivotal biomechanical transition zone between the rigid thoracic spine and the more mobile lumbar spine. Accurate morphometric data from this region are crucial for understanding human axial biomechanics and supporting forensic investigations. **Objective**: This study was designed to establish a normative multi-detector computed tomography (MDCT) database of the human thoracolumbar junction (T11–L2) in an adult population. Specifically, the primary objectives were to quantify normal vertebral and pedicle dimensions and to investigate the prevalence of congenital rib anomalies. The ultimate clinical goal is to translate these anatomical metrics into practical guidelines for spinal surgery and tools for forensic sex estimation. **Materials and Methods**: A retrospective analysis of 517 adults (277 men, 240 women) who received thoracic CT for various clinical indications was conducted. Morphometric parameters, including anterior, middle, and posterior vertebral heights; transverse and sagittal pedicle diameters; pedicle length; and transverse pedicle angle, were systematically measured. Costal variations, including hypoplastic and supernumerary ribs, were carefully recorded. **Results**: Vertebral and pedicle linear dimensions were generally larger in men than in women. The mean transverse pedicle diameter (TPD) increased progressively from T11 to L2. Significant sexual dimorphism in TPD was observed at the T11, L1, and L2 levels (*p <* 0.05), whereas both transverse (*p* = 0.168) and sagittal (*p* = 0.251) pedicle dimensions remained statistically comparable between sexes exclusively at the T12 transitional segment. The transverse pedicle angle (TPA) showed a consistent, increasing medial trajectory from T11 (~21°) to L2 (~28°) in both sexes, with no significant sex-based difference (*p* > 0.05). The overall prevalence of congenital rib variations was 18.57%. The most frequent structural anomaly was 12th-rib hypoplasia. **Conclusions**: This study provides a detailed morphometric baseline for the TLJ in an adult cohort (*n* = 517) of Turkish adults. The findings show that while absolute pedicle size and volumetric mass are influenced by sexually dimorphic physical geometry, the spatial trajectory and angulation of the pedicles (TPA) are determined by biomechanical principles that are independent of sex. In addition, pronounced sexual dimorphism and a high prevalence of unique costal variations were observed. These factors provide valuable biological markers that serve as reliable radiological fingerprints for forensic anthropologists who estimate sex from skeletal remains and identify unknown individuals.

## Simple Summary

The transitional zone of the spine between the mid-back and lowerback (the thoracolumbar junction) is highly prone to fractures and is a frequent site for major spinal surgeries. This study evaluated the detailed anatomical dimensions of these spinal bones and the presence of rib variations in 517 Turkish adults using computed tomography (CT) scans. Our findings show that while men generally have larger spinal bones than women, the dimensions at the exact transition point (the T12 level) become remarkably similar between genders. Additionally, nearly 18.57% of individuals have rib anomalies, such as a missing or underdeveloped 12th rib, which is significantly more common in women. These precise anatomical details provide a crucial roadmap for spine surgeons to plan safer procedures and avoid nerve damage or wrong-level surgeries, while also offering unique biological fingerprints to help forensic experts estimate sex and identify human remains.

## 1. Introduction

The thoracolumbar junction (TLJ), spanning T11 to L2, is the primary transition zone of the human axial skeleton. It marks a structural shift from the kyphotic, relatively immobile thoracic spine, stabilized by the rib cage and sternum, to the lordotic, highly dynamic lumbar spine [1,2]. Due to this unique architectural transition, the TLJ is subjected to considerable biomechanical stress and torsional forces. It is the most common anatomical site for acute spinal column injuries, predominantly encompassing traumatic fractures and osteoporotic compression fractures [3,4,5].Epidemiological data indicate that fractures at the thoracolumbar region account for approximately 50% to 60% of all spinal column injuries, presenting a substantial clinical and socioeconomic burden globally [3,4].Consequently, establishing a precise morphological rationale for this region is essential both for optimizing clinical interventions and improving patient outcomes following traumatic injuries [5].

The complex morphometry of the vertebral corpus and pedicles at the TLJ is characterized by rapid changes in size, shape, and spatial orientation. The vertebral pedicles function as the primary mechanical pathway, facilitating load transfer from the posterior elements to the vertebral body [6,7,8]. Because pedicle dimensions exhibit significant population-based variability [6,7] and specific angular differences [8], establishing normative morphometric profiles is clinically mandatory to guide safe transpedicular instrumentation and prevent surgical complications. High-resolution multidetector computed tomography (MDCT) with three-dimensional (3D) reconstruction is the most reliable imaging modality. It provides precise sub-millimeter morphometric data and helps safely navigate these complex bony architectures [9,10,11].

In addition to the intricate morphometry of the vertebrae, the TLJ is frequently associated with congenital anatomical variations. These include costal anomalies such as hypoplasia of the 12th rib, absent ribs, bifid ribs, and supernumerary lumbar or cervical ribs [9]. Understanding these structural variations has profound clinical and medicolegal implications. Clinically, it is vital for preventing surgical mislocalization. Additionally, these variations offer secondary utility in virtual anthropology as auxiliary biological markers for skeletal identification [10,11]. Despite the undeniable importance of regional morphometry, there remains a critical need for comprehensive normative databases that integrate detailed pedicle parameters, vertebral corpus dimensions, and costal variations in specific adult cohorts.

Although previous studies have investigated the thoracolumbar spine [1,6,12,13] or congenital rib anomalies [9] in the Turkish population, these anatomical parameters have predominantly been evaluated in isolation. A critical knowledge gap remains regarding the simultaneous, integrated assessment of pedicle dynamics, vertebral body dimensions, and costal variations. To address these clinical gaps, the present study was conducted to establish a standardized MDCT database of the human thoracolumbar junction. The specific objectives were defined as follows:

1. To quantify the normal linear and angular dimensions of the T11–L2 vertebrae and pedicles, thereby providing safe surgical baselines.
2. To determine the prevalence of congenital rib anomalies and evaluate their clinical impact on preventing wrong-level spine surgeries.
3. To analyze the sex-specific patterns of these morphological features for applications in forensic skeletal identification.

## 2. Materials and Methods

### 2.1. Study Design and Patient Selection

This retrospective observational study entailed a systematic review of high-resolution multidetector computed tomography (MDCT) images obtained between 1 January 2021, and 31 January 2025. The study protocol was approved by the Clinical Research Ethics Committee of the University of Health Sciences, Dr. Suat Seren Training and Research Hospital, Izmir, Turkey on 20 May 2026 (Protocol No: 2026/31–25).

To ensure a standardized and transparent patient selection process, the following clinical criteria were strictly applied:

#### 2.1.1. Inclusion Criteria

- Adult patients aged 18 years or older.
- Patients undergoing thoracic or abdominal MDCT scans during the specified study period.
- Scans performed for non-spinal and non-oncological clinical indications (such as interstitial lung disease, occupational pneumoconiosis screening, or pulmonary embolism protocols).

#### 2.1.2. Exclusion Criteria

- History of thoracic or spinal surgery, including chest wall interventions or prior spinal instrumentation.
- Pathological bone changes, including primary or metastatic spinal tumors.
- Congenital spinal deformities, major traumatic fractures, advanced degenerative disc disease, spondylolysis, or spondylolisthesis.
- Scoliosis with a Cobb angle exceeding 10 degrees.
- Suboptimal image quality due to motion artifacts or an incomplete field of view.

**Figure 1:**
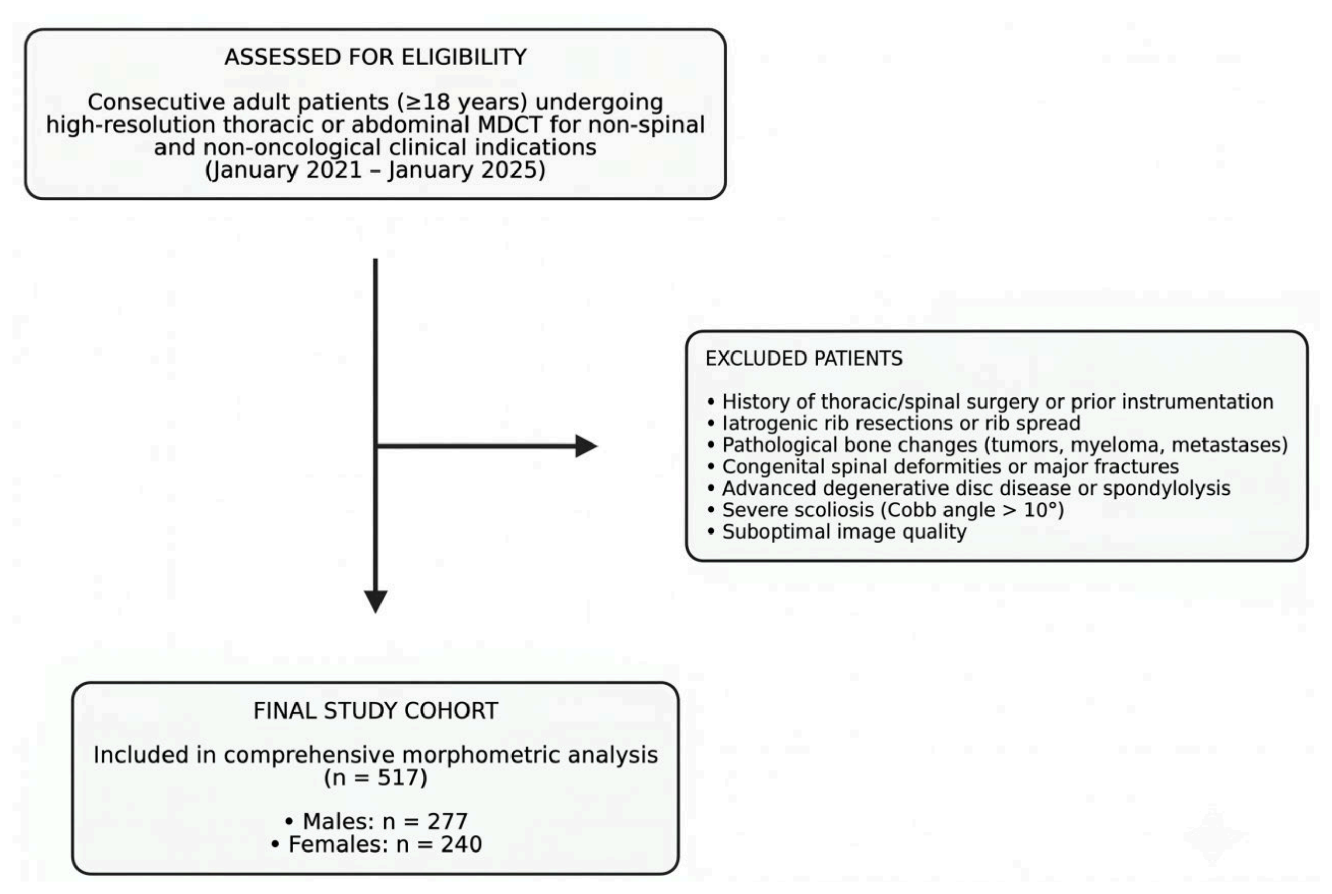
STROBE flow diagram illustrating the overview of the consecutive patient screening, exclusion pathways, and the final sample size allocation of the study cohort.

Following the systematic application of these criteria, a final cohort of 517 patients (277 men and 240 women) was established (Figure 1).

### 2.2. Imaging Protocol

All radiological data were obtained using a 64-slice multi-detector computed tomography (MDCT, Brilliance CT; Philips Medical Systems, Best, The Netherlands) operating at 120 kVp with automatic tube current modulation (AEC) and a range of 150–250 mA. Standardized scans were conducted with the patients in the supine position, capturing contiguous axial slices with a high-resolution thickness of 0.625 mm. The acquired raw datasets were subsequently exported to a dedicated medical Picture Archiving and Communication System (PACS) workstation for advanced post-processing. Multiplanar reformatted images and three-dimensional reconstructions were analyzed using a dedicated PACS workstation and advanced visualization software (IntelliSpace Portal, Version 11.1; Philips Medical Systems, Best, The Netherlands). To ensure maximum precision in delineating the cortical and trabecular boundaries, all multiplanar reformatted (MPR) images and three-dimensional (3D) reconstructions were evaluated in a standardized bone window setting (Window Width: 1500 HU; Window Level: 300 HU).

### 2.3. Morphometric Measurements

**Figure 2:**
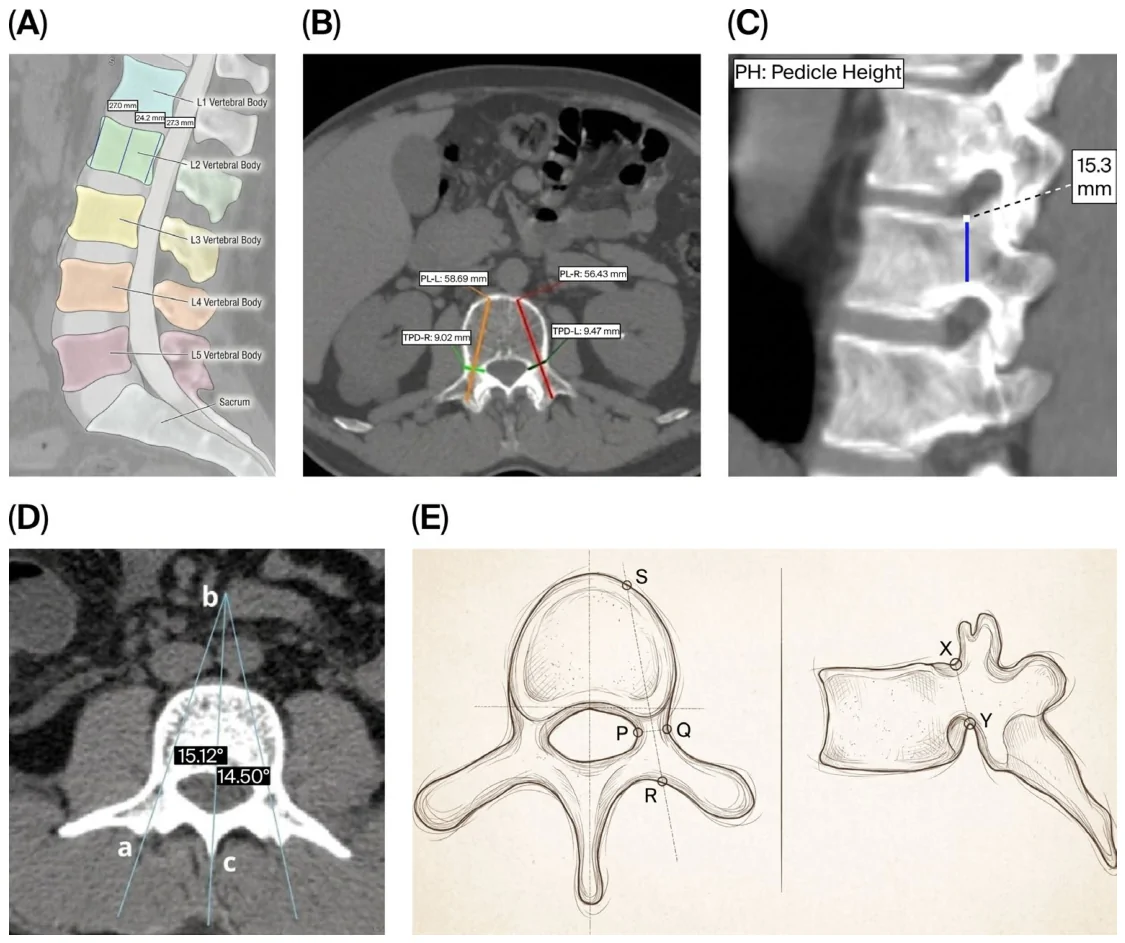
Axial and sagittal MDCT scans and schematic illustrations of the measurement techniques used to record pedicle parameters and vertebral corpus dimensions. (**A**) Sagittal CT reconstruction illustrating measurement of vertebral body height (as demonstrated on the L2 vertebral body using dashed blue lines to indicate the anterior, middle, and posterior corpus heights), with the lumbar vertebrae (L1–L5) and sacrum highlighted in distinct colors for anatomical orientation. (**B**) Axial CT image demonstrating morphometric measurements of a representative thoracolumbar vertebra. The orange and red lines indicate the right and left pedicle lengths (PL), respectively. The light and dark green lines represent the pedicle widths, also defined as the TPD. (**C**) Sagittal CT image demonstrating the measurement of the sagittal pedicle diameter (SPD). The blue vertical line indicates the maximum vertical height of the pedicle, defined as the SPD (pedicle height, PH). (**D**) Axial CT image illustrating the measurement of the TPA. Lines *a* and *c* delineate the central anatomical axes of the right and left pedicles, respectively, while line *b* represents the midsagittal axis of the vertebral body. The indicated angles (15.12° and 14.50°) correspond to the respective pedicle angulations. (**E**) Schematic anatomical drawings of a typical vertebra in axial (**left**) and sagittal (**right**) projections, detailing specific cortical landmarks (P, Q, R, S, X, Y) utilized as reference points for defining pedicle boundaries and standardized morphometric trajectories.

All morphometric procedures and anatomical reference points utilized in this study are not novel; rather, they strictly adhere to universally established and previously validated standard methodologies documented in the literature. Specifically, the linear and angular parameters of the thoracolumbar junction (TLJ) segments, namely T11, T12, L1, and L2, were systematically measured. The morphometric variables assessed included vertebral body parameters, namely the anterior, middle, and posterior corpus heights (ACH, MCH, and PCH), which were measured in the mid-sagittal plane to evaluate the sagittal profile in accordance with the measurement protocols described by Yerli et al. [1] and Güleç et al. [6]. To ensure strict standardization, the cortical landmarks illustrated in Figure 2E (P, Q, R, S, X, Y) were defined based on the classic geometric configurations established by Zindrick et al. [4] and Kim et al. [7]. Pedicle parameters consisted of the transverse pedicle diameter (TPD), measured at the narrowest pedicle isthmus between the outer cortical borders (points X and Y), and the sagittal pedicle diameter (SPD), defined as the maximum vertical height of the pedicle between the superior and inferior pedicle notches (points P and Q). Pedicle length (PL) was calculated as the distance from the posterior cortical surface of the articular process to the anterior vertebral body cortex (from point R to S), and the transverse pedicle angle (TPA) was measured between the central pedicle axis and the mid-sagittal axis of the vertebral body strictly following the landmark definitions established by Zindrick et al. [4] and Wu et al. [8]. Prior to final data extraction, this measurement framework was strictly validated through a preliminary calibration phase on a pilot sample of 30 random scans (completely excluded from the final cohort) to ensure that the software-based multiplanar alignment of all reference points was perfectly reproducible and fully standardized.

### 2.4. Evaluation of Anatomical Variations

CT images were systematically evaluated to identify congenital numerical and structural rib anomalies. The presence, absence, and precise lengths of the 12th rib were documented. Costal anomalies, including hypoplastic ribs (defined as less than 4 cm in length), supernumerary ribs (cervical or lumbar), bifid ribs, and foramen ribs, were recorded and classified by incidence and laterality.

### 2.5. Measurement Reliability

All morphometric measurements and evaluations of anatomical variations were performed by a single radiologist with 14 years of experience. To ensure the reproducibility and internal consistency of the data, fifty computed tomography scans were randomly selected and re-examined by the same radiologist four weeks after the initial analysis. The initial and repeated measurements were then compared using the intraclass correlation coefficient to determine intra-observer reliability.

### 2.6. Statistical Analysis

Statistical analyses were performed using SPSS software version 26.0 (IBM Corp., Armonk, NY, USA). Continuous data were expressed as mean values plus or minus standard deviations. The normal distribution of the metric variables, including vertebral body heights and pedicle measurements, was verified using the Kolmogorov–Smirnov and Shapiro–Wilk tests, along with visual analysis of Q-Q plots. The independent-samples *t*-test was used to compare continuous parameters between independent groups such as gender. To assess potential structural asymmetry between contralateral sides within the same individual, right and left dimensions were compared using the paired-samples *t*-test. Differences across the three chronological age groups were evaluated using a one-way analysis of variance (ANOVA), and the Tukey posthoc test was applied for multiple comparisons. Categorical data and the prevalence rates of congenital rib anomalies were compared using the Pearson chi-square test or Fisher’s exact test where appropriate. Intra-observer reliability for the repeated measurements was determined using the intraclass correlation coefficient. Statistical significance for all tests was defined as a *p*-value of less than 0.05.

It should be noted that the statistical comparisons across vertebral levels and parameters were conducted independently for each segment to provide clinically useful reference data. While it is acknowledged that multiple testing can increase the risk of type I error inflation, no formal multiplicity adjustment (e.g., Bonferroni correction) was applied. This approach was adopted to maintain sensitivity in detecting segmental anatomical variations that are of primary interest for spinal instrumentation. Consequently, the reported *p*-values were interpreted with caution, prioritizing findings that showed consistent anatomical trends across the thoracolumbar transition and were supported by robust clinical findings.

## 3. Results

### 3.1. Demographics and Vertebral Corpus Heights

The study cohort comprised 517 individuals with a mean age of 48.6 ± 16.4 years. All height parameters (ACH, MCH, and PCH) showed progressive increases in morphology from T11 to L2. Males exhibited significantly greater vertebral body dimensions than females at each level (*p <* 0.001) (Table 1).

#### Measurement Reliability

To evaluate the reproducibility of the morphometric framework, intra-observer reliability was rigorously tested using the Intraclass Correlation Coefficient (ICC). The repeated measurements performed on 50 randomly selected computed tomography scans demonstrated excellent internal consistency and reproducibility. The ICC values for all linear parameters (including transverse pedicle diameter, sagittal pedicle diameter, and vertebral heights) ranged from 0.912 to 0.965 (95% confidence interval: 0.895 to 0.978), indicating excellent agreement. Similarly, the ICC for angular measurements (transverse pedicle angle) was 0.898 (95% confidence interval: 0.872 to 0.924). All repeated intra-observer evaluations demonstrated high statistical reliability (*p <* 0.001).

Beyond statistical significance, the analysis reveals a consistent clinical disparity in vertebral corpus dimensions, with male vertebrae being approximately 9.1% to 11.9% larger than female counterparts across all levels. Calculation of Cohen’s d effect sizes further confirmed a ‘large’ practical effect (d > 0.8) for all vertebral height parameters. These findings carry significant biomechanical implications; the sex-specific dimorphism in vertebral height necessitates gender-stratified planning for spinal instrumentation, such as pedicle screw length and vertebral body replacement sizing, to avoid implant-bone mismatch and associated complications.

### 3.2. Morphometric Analysis of Pedicle Diameters and Asymmetry

**Figure 3:**
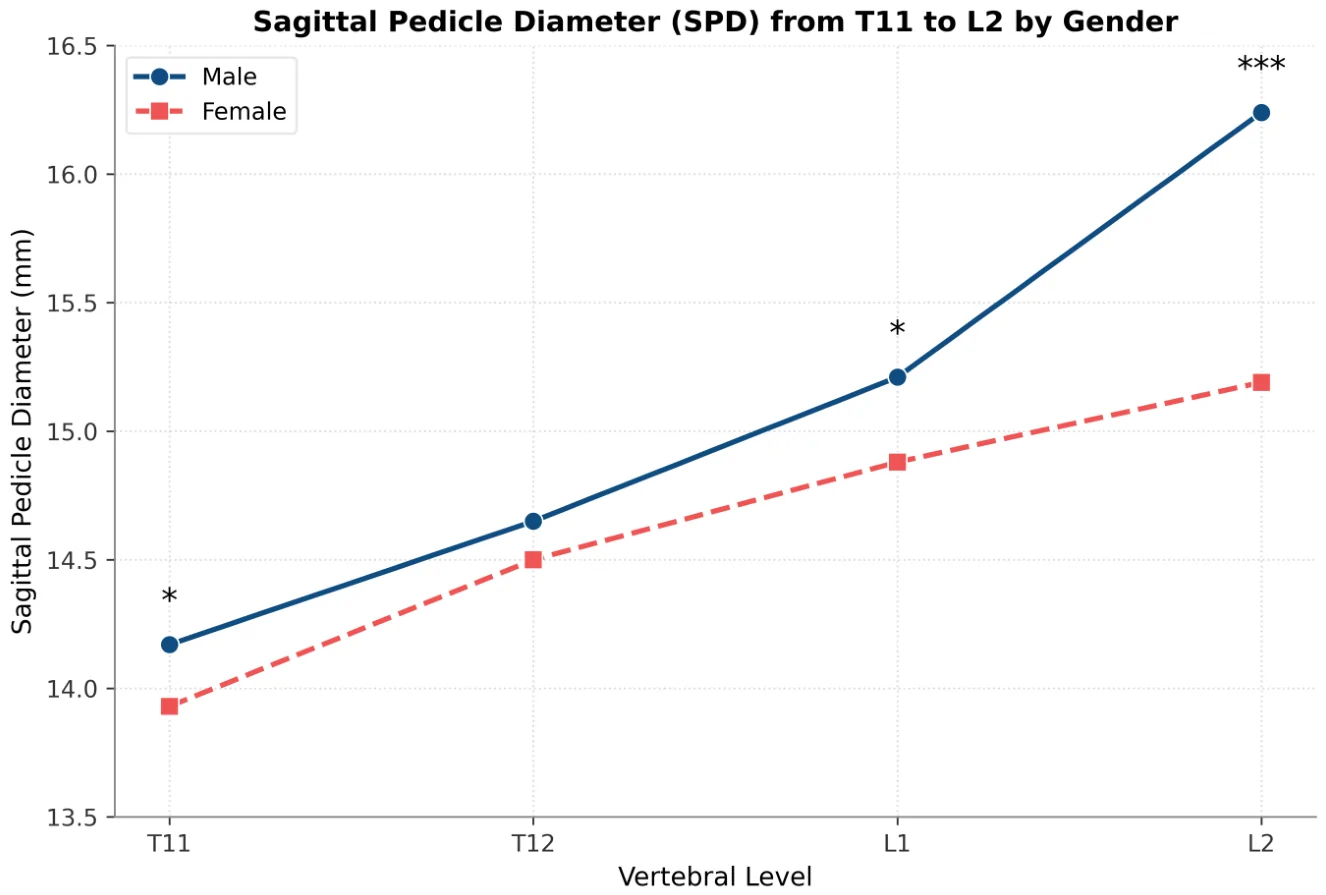
Sagittal pedicle diameter (SPD) from T11 to L2 by gender. Males have significantly larger SPDs at T11, L1, and L2, whereas T12 values are similar between sexes. (* *p <* 0.05, *** *p <* 0.001).

Both TPD and SPD demonstrated a progressive craniocaudal increase from T11 to L2. Significant sexual dimorphism in TPD was observed at T11, L1, and L2 (*p <* 0.05), with no difference between sexes exclusively at the T12 segment (*p* = 0.168). In contrast, SPD exhibited significant sexual dimorphism across the thoracolumbar junction (at T11, L1, and L2), except at the T12 transitional segment, where vertical dimensions remained statistically comparable between sexes (*p* = 0.251) (Table 2, Figure 3). A specific evaluation of the T11 segment revealed that nearly 11% of the patients (*n* = 57) possessed a TPD of less than 6 mm, presenting a potential anatomical bottleneck.

Furthermore, comprehensive paired samples *t*-tests confirmed high morphometric concordance and no statistically significant asymmetry between the right and left pedicles. Measurements of the TPD across all evaluated thoracolumbar segments revealed no statistically significant right-left asymmetry, with the tight 95% confidence intervals further demonstrating the precision of these minimal variations (mean differences ranging from 0.04 to 0.09 mm for TPD, and 0.17 mm for PL; *p >* 0.05) (Table 3).

### 3.3. Age-Stratified Morphometric Data

To address the effects of degenerative changes and skeletal maturation, the cohort was categorized into three distinct age groups: 18–35 years (*n* = 198), 36–55 years (*n* = 176), and >55 years (*n* = 143). This stratification represents a methodological approach well supported by Köken et al. [12]. While minor morphological differences were observed among these age groups, one-way ANOVA demonstrated no statistically significant age-related differences in TPD within the same sex across the evaluated segments (*p >* 0.05). For instance, the mean male TPD at the L1 level was 9.45 ± 1.55 mm in the youngest cohort (18–35 years) and increased slightly to 9.62 ± 1.72 mm in the oldest cohort (>55 years); however, this difference was not statistically significant (*p* = 0.185). These findings clearly demonstrate the morphological stability of the pedicle width throughout adulthood once skeletal maturity is reached (Table 4).

### 3.4. Prevalence of Congenital Costal Anomalies

**Figure 4:**
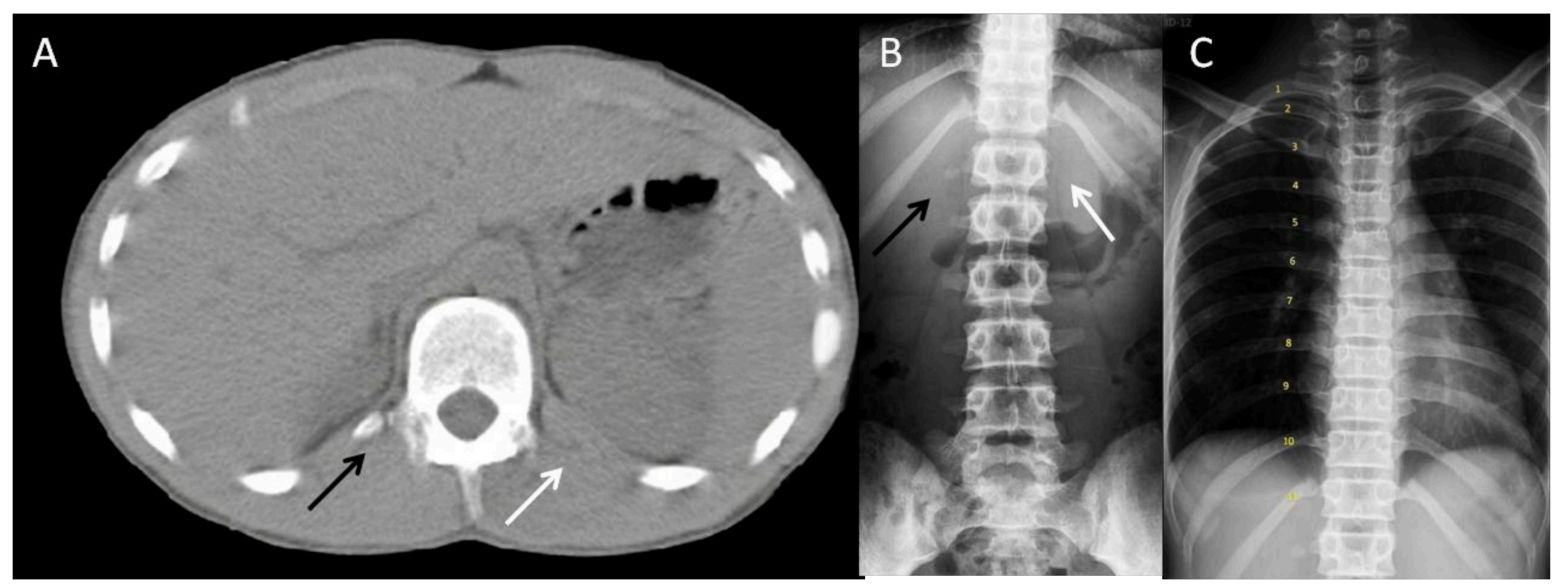
Computed tomography and radiographic evaluation of 12th-rib variations. Panels (**A**,**B**) are obtained from a single representative patient; the axial CT image (**A**) and the correlated anteroposterior radiograph (**B**) demonstrate a hypoplastic right 12th rib (indicated by black arrows) and a concomitantly absent left 12th rib (indicated by white arrows). Panel (**C**) is obtained from a separate patient, showing a frontal radiograph highlighting bilateral costal variations for comparison.

**Figure 5:**
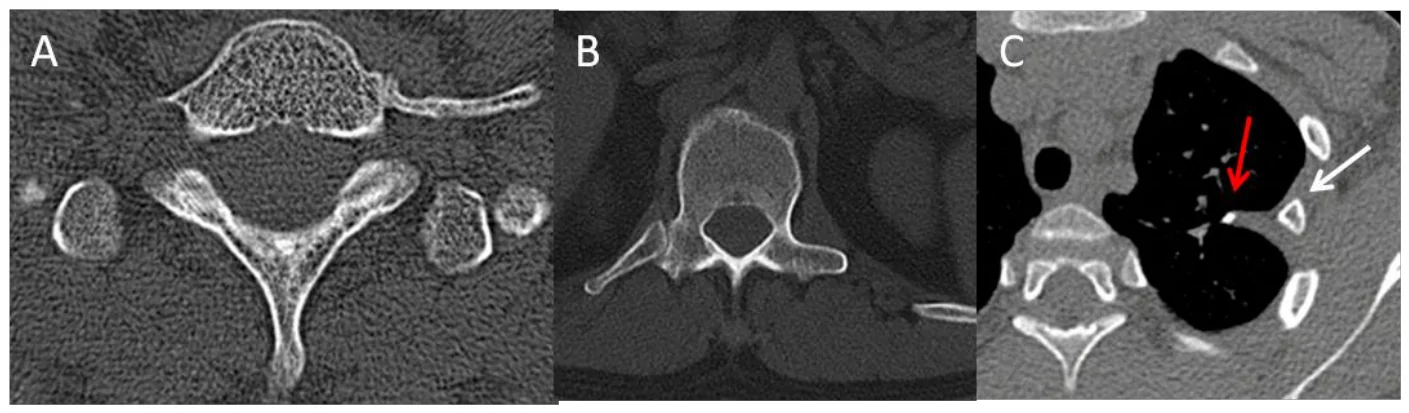
Axial CT images of accessory and supernumerary ribs. (**A**) A cervical rib. (**B**) A lumbar rib. (**C**) Thoracic CT demonstrating a supernumerary rib (red arrow) arising from the posterior aspect of a short left third rib (white arrow) and coursing inferolaterally toward the pulmonary parenchyma.

**Figure 6:**
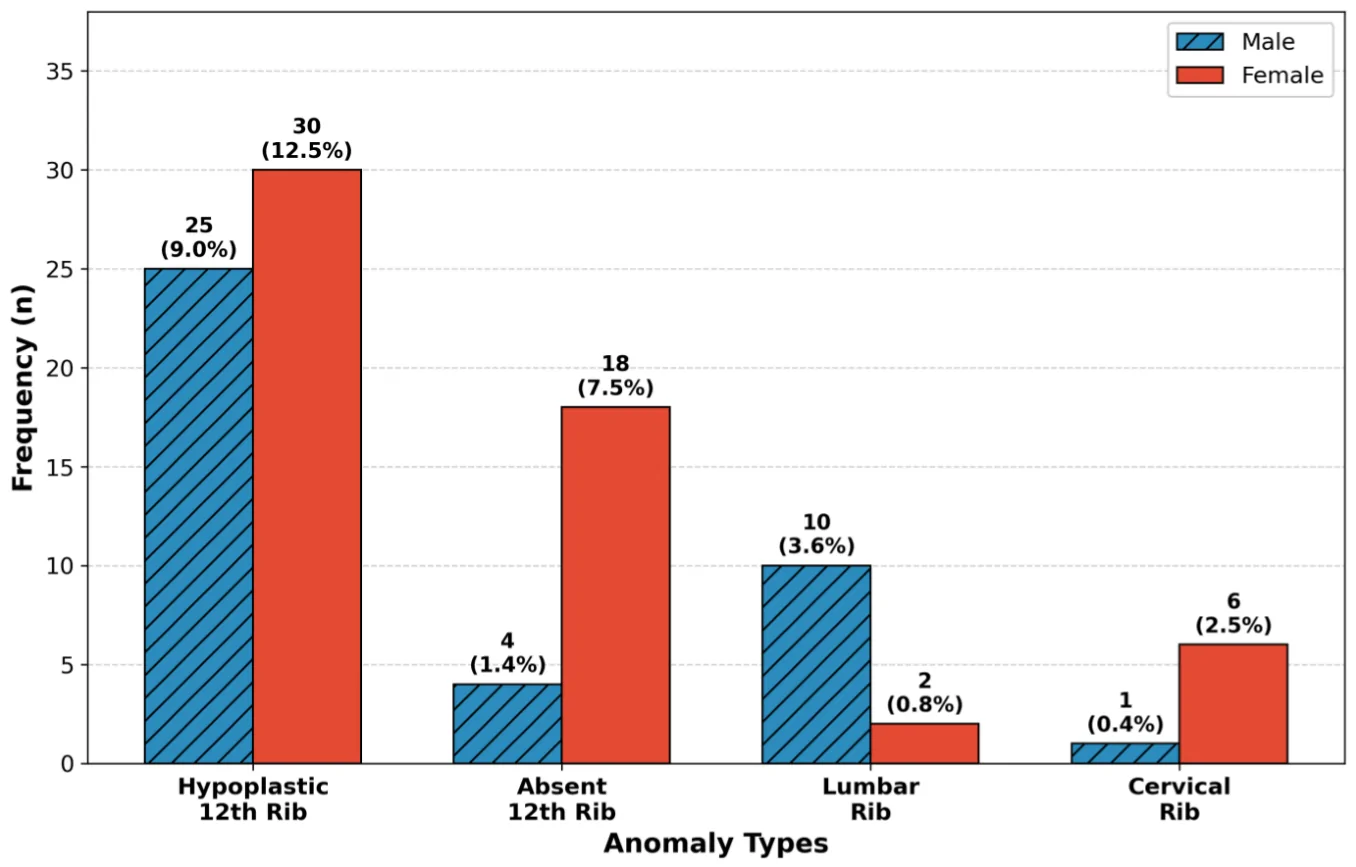
Sex-specific distribution and prevalence of congenital costal anomalies. The clustered bar chart illustrates the frequencies of congenital rib anomalies categorized by sex. Note the statistically significant higher prevalence of total rib anomalies in females (*p* = 0.013) and the specific predilection for 12th-rib absence (*p* = 0.001), emphasizing the potential of these structural variations as sex-diagnostic markers in forensic anthropology.

The overall prevalence of rib anomalies was 18.57%, representing 96 of 517 individuals. Pearson Chi-square analysis demonstrated a statistically significant difference in overall anomaly distribution between sexes, with a notably higher prevalence observed in females, at 23.33% (56 of 240), compared to males, at 14.44% (40 of 277) (Chi-square value = 6.143, *p*-value = 0.013) Specifically, the complete absence of the 12th rib showed a profound female predilection (*p*-value = 0.001) (Figure 4), and cervical ribs were also significantly more frequent in females (*p*-value = 0.045) (Figure 5). In contrast, lumbar ribs exhibited a statistically significant male dominance (*p*-value = 0.036). Hypoplastic 12th ribs remained the most frequent structural variation across the entire cohort, though its specific distribution between genders did not achieve statistical significance (*p*-value = 0.322) (Table 5, Figure 6).

## 4. Discussion

This study demonstrates that both vertebral and pedicle linear dimensions exhibit a progressive craniocaudal increase across the thoracolumbar transition zone, accompanied by a well-defined sexual dimorphism at most levels. However, a unique anatomical equalization is observed exclusively at the T12 segment, where pedicle dimensions remain statistically comparable between sexes. In contrast, the spatial trajectory and medial angulation of the pedicles remain highly stable and consistent between genders across the entire transition zone. Furthermore, the cohort exhibits a high regional prevalence of congenital costal variations, characterized predominantly by twelfth-rib hypoplasia and complete absence, both of which display distinct, statistically validated sex-specific distribution patterns.

### 4.1. Anatomical, Biomechanical, and Surgical Significance of Pedicle Morphology

The morphometric evaluation revealed a progressive craniocaudal widening of the transverse pedicle diameter (TPD) from T11 to L2, accompanied by consistently larger sagittal pedicle diameters (SPD). This vertical, elliptical configuration reflects the physiological adaptation of the axial skeleton to increasing mechanical loads, in profound agreement with Köken et al. [12] and Düzkalır et al. [13]. Regarding sexual dimorphism, the analysis yielded highly specific segmental results; while dimensions were significantly larger in males at T11, L1, and L2, the T12 transitional segment showed no sex difference in absolute measures. This specific segmental equalization highlights the unique role of T12 in axial load transfer, differing from the broader, pan-lumbar sexual dimorphism reported elsewhere [14,15].

The clinical utility of these dimensions becomes evident at the T11 level, where the narrow TPD acts as a critical bottleneck during transpedicular screw placement. Surgical literature establishes that pedicle width is the primary limiting factor for selecting safe screw diameters [16,17], and a mismatch directly increases the risk of cortical breach [18]. Medial or lateral perforations can lead to severe complications, with medial breaches being particularly dangerous due to the risk of dural tears, cerebrospinal fluid leaks, and direct neural injury [19].

Beyond reporting mean dimensions, a clinically actionable risk-threshold model regarding pedicle morphology was established in this study. Given that a substantial proportion of the cohort demonstrated narrow pedicle diameters particularly at the T11 level, these morphometric constraints necessitate a rigorous segment-specific risk assessment to prevent pedicle cortical perforation during transpedicular instrumentation.

### 4.2. TPA Dynamics and Contralateral Symmetry

The spatial orientation of the pedicle, defined by the transverse pedicle angle (TPA), is a critical determinant of a safe screw trajectory during posterior instrumentation [20]. The data demonstrate that pedicles maintain a consistent, progressively increasing medial trajectory in both sexes, steadily increasing from approximately 21 degrees at T11 to nearly 28 degrees at L2. Unlike linear dimensions, this angular stability indicates that spatial orientation is dictated by shared, sex-independent biomechanical transition principles.

In terms of laterality, the paired analyses confirmed the absence of statistically significant structural asymmetry between the right and left pedicles across all evaluated segments. This structural balance aligns with the normative patterns documented by Wu et al. [8] and Senturk [21]. However, from a precise surgical standpoint, it is critical to acknowledge that the presence of even sub-millimeter mean differences (ranging from 0.04 to 0.09 mm in TPD) indicates that minor individual variations exist. While these minor differences are statistically negligible on a population-based level, they can become clinically relevant in highly restrictive surgical corridors, such as the T11 segment. In patients with borderline pedicle dimensions, a sub-millimeter lateral discrepancy could theoretically affect the tolerance margin for safe transpedicular screw trajectory. Therefore, despite the predictable cohort-level bilateral consistency documented in this study, spine surgeons should continue to conduct individualized, side-specific multiplanar CT evaluations prior to instrumentation to eliminate any localized risks of cortical wall violation. This predictable medial convergence is highly advantageous. While advanced three-dimensional intraoperative navigation systems, such as the O-arm, significantly improve screw accuracy [19], their routine use is often limited by high healthcare costs and prolonged operative times [22]. In resource-limited settings where surgeons rely on free-hand techniques or conventional two-dimensional fluoroscopy, the sex-independent predictability of the TPA provides a highly reliable anatomical baseline [23], serving as a natural ‘safe corridor’ for transpedicular fixation [24].

### 4.3. Costal Variations and the 12th Rib

To evaluate the clinical and forensic significance of the observed costal anomalies, comprehensive comparative statistical analyses were performed between sexes (Table 5). While the overall prevalence of rib anomalies was 18.57% in the Turkish cohort, the quantitative analysis demonstrated a statistically significant sex-based difference in distribution (*p*-value less than 0.05 via Chi-square test), with females exhibiting a higher overall prevalence of these variations. Specifically, the strong female correlation with a completely absent 12th rib (*p*-value = 0.001) and the explicit male association with lumbar ribs (*p*-value = 0.036) confirm that congenital costal variations are not merely incidental or purely descriptive findings, but instead possess distinct, statistically validated sexually dimorphic patterns. Twelfth-rib hypoplasia was the most frequently observed structural anomaly across the entire study population, followed by the complete absence of the 12th rib, which aligns with the structural baseline trends previously noted by Oner et al. [9].

Beyond these demographic distributions, this high prevalence of congenital costal anomalies carries profound clinical and surgical importance. Accurate identification of vertebral levels is a fundamental prerequisite for any spinal intervention, and anomalous spinal anatomy is a well-documented risk factor for localization errors. When the 12th rib is rudimentary or absent, counting vertebrae from caudal to cranial rather than from cranial to caudal during preoperative planning and intraoperative fluoroscopy often yields conflicting results. This discrepancy significantly increases the risk of operating on the wrong adjacent segment, leading to wrong-level spine surgery, which remains a devastating and preventable “never event” with severe physical and medicolegal consequences [25,26,27]. Furthermore, as highlighted by Korkmaz and Özevren [25], the 12th thoracic vertebra and its associated structures reside in exceptionally close proximity to major retroperitoneal vascular structures, such as the thoracic aorta. Atypical costal architecture at this level can significantly alter standard surgical landmarks. Therefore, spine surgeons should not rely solely on standard anteroposterior radiographs for level localization. A detailed preoperative evaluation using cross-sectional computed tomography to trace the entire rib cage is strongly recommended, thereby ensuring accurate vertebral numbering and eliminating the risk of catastrophic iatrogenic injuries [28,29]. In addition to these critical surgical safety implications, these statistically validated prevalence rates provide highly reliable biological reference points in forensic investigations. The observed female predilection for 12th-rib absence suggests that this structural variation can serve as a reliable discriminatory indicator in sex estimation models, especially when integrated with traditional pelvic or cranial markers. In the fields of clinical anatomy, forensic anthropology, and medicolegal profiling, our quantitative findings substantiate the use of the 12th rib as a robust biological marker. These variations effectively transform simple structural anomalies into objective radiological fingerprints, significantly increasing the mathematical accuracy of sex estimation and personal identification protocols from compromised skeletal remains [10,11].

### 4.4. Forensic and Anthropological Implications

In addition to their clinical utility, the morphometric data and anatomical variations documented in this study hold substantial forensic and anthropological significance. Forensic identification protocols increasingly rely on radiological markers to establish biological profiles, particularly when analyzing incomplete or degraded skeletal remains. As demonstrated by Ekizoglu et al. [10], virtual morphometry of the lumbar vertebrae, specifically the L1 segment, provides highly reliable parameters for sex estimation in the Turkish population. The findings significantly expand upon this by detailing the pronounced sexual dimorphism present not only in vertebral body heights but also in the transverse and sagittal pedicle diameters across the entire T11–L2 transition zone. Furthermore, Rohmani et al. [11] highlighted the robust utility of human vertebrae in systematic sex estimation. The highly specific, segment-dependent dimorphism observed in our cohort—such as the unique equalization of pedicle dimensions exclusively at the T12 level—adds a granular layer of population-specific data that can refine these anthropological profiling techniques.

Moreover, the high prevalence (18.57%) and sex-specific distribution of congenital rib anomalies, consistent with the regional patterns noted by Oner et al. [9], serve as unique individual biological identifiers. In medicolegal death investigations, where antemortem and postmortem radiographic comparisons are paramount, identifying an asymptomatic hypoplastic or absent 12th rib can provide definitive corroborating evidence for positive human identification. Finally, establishing an accurate population-specific baseline for vertebral heights is critical for evaluating traumatic vertebral fractures in forensic medical reports, ensuring that pathological compressions are accurately distinguished from natural morphological wedging [5]. Notably, recent advancements in virtual anthropology heavily emphasize the indispensable role of MDCT in establishing these population-specific skeletal standards, further reinforcing the medicolegal value of our normative database [30,31].

### 4.5. Toward Clinical Decision Support: Proposing New Diagnostic Thresholds

The findings allow for the derivation of practical decision-support tools beyond simple descriptive statistics. First, a ‘Pedicle Safety Threshold’ (PST) is defined at the T11 level. Given that 11% of the adult cohort presented with a TPD <6 mm—a threshold frequently associated with an unacceptably high risk of cortical breach during 6.5 mm pedicle screw insertion—it is proposed that any patient undergoing TLJ instrumentation with a TPD <6 mm be automatically considered for alternative fixation strategies, such as smaller-diameter screws (4.5–5.5 mm) or extrapedicular techniques, to ensure cortical integrity. Second, the analysis of congenital rib variations provides the basis for a ‘Standardized Rib-Tracing Protocol’. In light of the 18.57% prevalence of costal anomalies, it is suggested that reliance on standard AP radiographs for vertebral localization is insufficient. A mandatory preoperative cross-sectional CT ‘rib-tracing’ protocol is advocated for all TLJ procedures, particularly in female patients, who showed a statistically higher predilection for these localization-distorting anomalies (*p* = 0.013). Implementing these thresholds and protocols shifts these morphometric data from a purely descriptive atlas to a direct clinical decision-support framework aimed at preventing surgical ‘never events’.

### 4.6. Limitations

Although this investigation establishes a comprehensive, high-resolution multidetector computed tomography morphometric baseline of the thoracolumbar junction within a substantial adult cohort, several native limitations warrant consideration. First, the retrospective, single-center design conducted at a specialized tertiary thoracic center may introduce inherent selection bias, potentially limiting the direct generalizability of these normative values to more heterogeneous national or global populations. Second, due to the anonymized nature of the radiological database, critical somatometric data—including body mass index, total height, and specific physical workload history—were unavailable, which precluded the assessment of potential correlations between individual physical geometry and absolute vertebral dimensions. Furthermore, while the cohort was stratified across distinct age groups to evaluate morphology, the static, cross-sectional nature of the imaging protocol did not allow for the prospective monitoring of longitudinal osteoporotic remodeling or subclinical micro-structural alterations over time. Additionally, the strict exclusion of individuals with advanced spinal pathologies, severe degenerative disc disease, and scoliosis exceeding a ten-degree Cobb angle means these data reflect pristine anatomy and may not accurately represent the compromised structural morphology frequently encountered in geriatric or severely deformed clinical populations. Finally, all multi-detector computed tomography datasets were acquired in the supine position, capturing a static, non-weight-bearing state of the axial skeleton; consequently, these measurements do not account for the dynamic spatial alterations, torsional stresses, or biomechanical strain variations that occur during physiological upright posture and multi-axial spinal kinetics.

Regarding the age-stratified analysis (Table 4), it must be acknowledged that the reliance on *p*-values alone—which were non-significant across all segments—may be limited by the absence of confidence intervals or effect size measures. While the study population (*n* = 517, comprising 277 males and 240 females) is statistically defined for descriptive morphometry, the study was not explicitly powered to detect subtle, clinically minor differences in pedicle diameter across age groups. Nevertheless, the observed mean differences across cohorts were consistently below 0.2 mm, which is below the threshold of clinical relevance for surgical screw selection. This consistency suggests that any age-related change in transverse pedicle diameter (TPD) within this Turkish population is likely negligible.

### 4.7. Future Research Directions

To build upon the normative baseline established in this investigation, several distinct avenues for future research are warranted. First, prospective, multicenter collaborative studies encompassing larger, multi-ethnic cohorts are essential to validate the generalizability of these morphometric thresholds across diverse populations beyond the evaluated Turkish cohort. Second, future investigations should integrate comprehensive somatometric and clinical datasets—such as body mass index (BMI), exact standing height, bone mineral density (BMD), and long-term physical workload histories—to evaluate their precise correlations with regional vertebral and pedicle geometry. Third, incorporating weight-bearing or dynamic imaging modalities, such as upright cone-beam computed tomography (CBCT), would provide critical functional insights into the live spinal kinetics, torsional stresses, and biomechanical strain variations in the thoracolumbar junction during physiological posture. Finally, leveraging deep learning and artificial intelligence (AI) networks for automated, high-throughput morphometric segmentation and automated sex estimation models represents a promising frontier to actively enhance and accelerate objective diagnostic workflows in both clinical spinal surgery and virtual forensic anthropology.

## 5. Conclusions

In conclusion, this study establishes a rigorous, population-specific MDCT morphometric baseline for the human thoracolumbar junction. The data demonstrate that while physical bone volumes and pedicle dimensions are heavily influenced by sexual dimorphism, the spatial trajectory and medial angulation of the pedicles remain uniform across sexes due to shared biomechanical principles. Additionally, the high prevalence of congenital costal variations, particularly in females, underscores a significant anatomical risk factor for wrong-level spinal surgeries. Rather than relying on standard radiographs, implementing mandatory preoperative cross-sectional rib-tracing protocols and utilizing segment-specific pedicle safety thresholds will actively prevent surgical complications and substantially improve the accuracy of forensic skeletal identification.

## Figures and Tables

**Table 1:** Vertebral Corpus Heights in the Study Cohort (Mean ± SD [95% CI] mm).

Level	Parameter	Male (Mean ± SD)	Female (Mean ± SD)	95% CI (Male/Female)	Diff (%)	*p*-Value
**T11**	ACH	21.43 ± 1.87	19.22 ± 1.54	[21.21–21.65]/[19.02–19.42]	+11.5%	<0.001
	MCH	22.11 ± 1.65	20.03 ± 1.48	[21.91–22.31]/[19.84–20.22]	+10.4%	<0.001
	PCH	23.08 ± 1.92	20.81 ± 1.63	[22.85–23.31]/[20.60–21.02]	+10.9%	<0.001
**T12**	ACH	23.61 ± 2.14	21.09 ± 1.72	[23.35–23.87]/[20.87–21.31]	+11.9%	<0.001
	MCH	24.04 ± 1.88	21.55 ± 1.66	[23.82–24.26]/[21.34–21.76]	+11.5%	<0.001
	PCH	24.83 ± 2.07	22.19 ± 1.85	[24.58–25.08]/[21.95–22.43]	+11.9%	<0.001
**L1**	ACH	24.78 ± 2.05	22.56 ± 1.91	[24.53–25.03]/[22.31–22.81]	+9.8%	<0.001
	MCH	25.12 ± 1.76	23.02 ± 1.64	[24.91–25.33]/[22.81–23.23]	+9.1%	<0.001
	PCH	25.94 ± 1.98	23.57 ± 1.82	[25.70–26.18]/[23.33–23.81]	+10.0%	<0.001
**L2**	ACH	26.31 ± 2.21	23.85 ± 1.95	[26.05–26.57]/[23.60–24.10]	+10.3%	<0.001
	MCH	26.55 ± 1.98	24.11 ± 1.78	[26.31–26.79]/[23.88–24.34]	+10.1%	<0.001
	PCH	27.12 ± 2.15	24.68 ± 1.92	[26.86–27.38]/[24.43–24.93]	+9.9%	<0.001

Abbreviations: ACH, anterior corpus height; MCH, middle corpus height; PCH, posterior corpus height; SD, standard deviation, CI: confidence interval.

**Table 2:** Pedicle Parameters at the Thoracolumbar Junction.

Level	Parameter	Male (Mean ± SD)	Female (Mean ± SD)	*p*-Value
**T11**	TPD (mm)	7.62 ± 1.35	7.21 ± 1.14	<0.001
	SPD (mm)	14.17 ± 1.42	13.93 ± 1.31	0.046
	TPA (°)	21.18 ± 3.20	20.98 ± 2.50	0.424
**T12**	TPD (mm)	8.46 ± 1.42	8.28 ± 1.53	0.168
	SPD (mm)	14.65 ± 1.51	14.50 ± 1.44	0.251
	TPA (°)	23.45 ± 3.73	23.35 ± 2.21	0.706
**L1**	TPD (mm)	9.53 ± 1.64	8.88 ± 1.72	<0.001
	SPD (mm)	15.21 ± 1.43	14.88 ± 1.55	0.012
	TPA (°)	25.55 ± 5.33	25.33 ± 4.47	0.610
**L2**	TPD (mm)	10.08 ± 1.71	9.72 ± 1.83	0.021
	SPD (mm)	16.24 ± 1.72	15.19 ± 1.84	<0.001
	TPA (°)	27.79 ± 5.55	27.32 ± 4.78	0.303

Abbreviations: TPD, transverse pedicle diameter; SPD, sagittal pedicle diameter; TPA, transverse pedicle angle; SD, standard deviation.

**Table 3:** Comparative Analysis of Right and Left Pedicle Morphometric Parameters.

Level	Parameter	Right Pedicle (Mean ± SD)	Left Pedicle (Mean ± SD)	Mean Difference (95% CI)	Mean Difference	*p*-Value
**T11**	TPD (mm)	7.51 ± 1.22	7.42 ± 1.28	0.09 (−0.02–0.20)	0.09	0.31
	PL (mm)	41.25 ± 2.14	41.08 ± 2.20	0.17 (−0.13–0.19)	0.17	0.41
**T12**	TPD (mm)	8.32 ± 1.45	8.28 ± 1.50	0.04 (−0.09–0.17)	0.04	0.58
**L1**	TPD (mm)	9.25 ± 1.60	9.21 ± 1.65	0.04 (−0.11–0.19)	0.04	0.65
**L2**	TPD (mm)	9.93 ± 1.70	9.89 ± 1.80	0.04 (−0.12–0.20)	0.04	0.78

Abbreviations: TPD, transverse pedicle diameter; PL, pedicle length; SD, standard deviation; CI: confidence interval; Paired samples *t*-test, *p* < 0.05 is considered significant.

**Table 4:** Age-stratified analysis of transverse pedicle diameter (TPD) across the adult lifespan.

Level	Gender	Group 1 (18–35)	Group 2 (36–55)	Group 3 (>55)	Mean Diff (95% CI)	*p*-Value
**T11**	Male	7.55 ± 1.25	7.62 ± 1.30	7.68 ± 1.42	0.07 (−0.08–0.22)	0.210
	Female	7.15 ± 1.10	7.21 ± 1.15	7.28 ± 1.18	0.06 (−0.07–0.19)	0.450
**T12**	Male	8.38 ± 1.35	8.46 ± 1.40	8.52 ± 1.45	0.07 (−0.10–0.24)	0.305
	Female	8.23 ± 1.42	8.28 ± 1.50	8.33 ± 1.55	0.05 (−0.11–0.21)	0.112
**L1**	Male	9.45 ± 1.55	9.53 ± 1.60	9.62 ± 1.72	0.08 (−0.12–0.28)	0.185
	Female	8.80 ± 1.65	8.88 ± 1.70	8.95 ± 1.81	0.07 (−0.13–0.27)	0.312
**L2**	Male	9.98 ± 1.65	10.08 ± 1.72	10.18 ± 1.80	0.10 (−0.13–0.33)	0.245
	Female	9.65 ± 1.75	9.72 ± 1.82	9.80 ± 1.88	0.07 (−0.16–0.30)	0.410

Abbreviations: CI: confidence interval.

**Table 5:** Sex-Specific Distribution and Prevalence of Congenital Rib Anomalies.

Anomaly Type	Total Cohort (*n* = 517), *n* (%)	Male (*n* = 277), *n* (%)	Female (*n* = 240), *n* (%)	Chi-Square (X^2^)	*p*-Value
Hypoplastic 12th Rib	55 (10.64%)	25 (9.03%)	30 (12.50%)	0.982	0.322
Absent 12th Rib	22 (4.26%)	4 (1.44%)	18 (7.50%)	11.231	0.001
Lumbar Rib	12 (2.32%)	10 (3.61%)	2 (0.83%)	4.412	0.036
Cervical Rib	7 (1.35%)	1 (0.36%)	6 (2.50%)	4.021	0.045
**Overall Anomalies**	96 (18.57%)	40 (14.44%)	56 (23.33%)	6.143	0.013

*p*-values were calculated using the Chi-square test with *p* < 0.05 considered statistically significant.

## Data Availability

The datasets generated and analyzed during the current study are not publicly available due to institutional patient privacy regulations. However, the anonymized data are available from the corresponding author upon reasonable academic request.
